# Re-expansion of vertebral compression fractures in patients with multiple myeloma with percutaneous vertebroplasty using spinejack implants: a preliminary and retrospective study

**DOI:** 10.3389/fsurg.2023.1121981

**Published:** 2023-05-23

**Authors:** Claudio Pusceddu, Eliodoro Faiella, Daniele Derudas, Nicola Ballicu, Luca Melis, Stefano Zedda, Salvatore Marsico

**Affiliations:** ^1^ Division of Interventional Radiology, Department of Oncological Radiology, Ocological Hospital “A. Businco”, Regional Referral Center for Oncologic Diseases, Cagliari, Italy; ^2^ Department of Radiology, Sant'Anna Hospital, San Fermo Della Battaglia, Italy; ^3^ Department of Hematology, Businco Hospital, Cagliari, Italy; ^4^ Division of Interventional Radiology, Department of Oncological Radiology, Ocological Hospital “A. Businco”, Regional Referral Center for Oncologic Diseases, Cagliari, Italy; ^5^ Department of Oncological Radiology, Oncological Hospital “A. Businco”, Regional Referral Center for Oncological Diseases, Cagliari, Italy; ^6^ Department of Radiology, Hospital del Mar, Barcelona, Spain

**Keywords:** expandable titanium spineJack implants, interventional radiology, percutaneous therapies, multiple myeloma, vertebroplasty

## Abstract

**Objective:**

To retrospectively evaluate the feasibility and effectiveness of vertebroplasty using Spinejack implantation for the treatment and stabilization of painful vertebral compression fractures, in patients diagnosed with Multiple Myeloma (MM), to allow both an effective pain reduction and a global structural spine stabilization.

**Materials and Methods:**

From July 2017 and May 2022 thirty-nine patients diagnosed MM, with forty-nine vertebral compression fractures underwent percutaneous Vertebroplasty using Spinejack Implants. We analyzed the feasibility and complications of the procedure, the decrease in pain using visual analogue scale (VAS) and Functional Mobility Scale (FMS).

**Results:**

The technical success rate was 100%. No procedure-related major complications or death occurred. In the 6-month follow-up, the mean VAS score decreased from 5.4 ± 1.0 to 0.2 ± 0.5 with a mean reduction of 96.3%. FMS decreased from 2.3 ± 0.5 vs. 1.2 ± 0.4 with a mean reduction of −47.8%. There were no major complications related to incorrect positioning of the Expandable Titanium SpineJack Implants. In five patients, a cement leak was observed with no associated clinical manifestations. The average length of hospital stay was 6–8 Hours6.6 ± 1.2 h. No new bone fractures or local disease recurrence occurred during a median contrast-enhanced CT follow-up of 6 months.

**Conclusions:**

Our results suggest that vertebroplasty, using Spinejack implantation for the treatment and stabilization of painful vertebral compression fractures, secondary to Multiple Myeloma is a safe and effective procedure with long - term pain relief and restoration of vertebral height.

##  Introduction

Multiple myeloma (MM) is a clonal plasma cell proliferative disorder characterized by the abnormal increase of monoclonal immunoglobulins that can ultimately evolve to specific end-organ damage.

MM represents about 1.8% of all new cancer cases diagnosed in the United States each year with a median age of about 70 years and is slightly more commonly seen in males than females (1.4:1).

Bone lesions are seen in 80% of patients with MM which are complicated frequently by skeletal-related events (SRE) such as hypercalcemia, bone pain, pathological fractures, vertebral collapse, and spinal deformities.

The most commonly used therapies in treatment of MM are radiotherapy (RT), antiresorptive therapies (bisphosphonates, denosumab) and Systemic Anti-Myeloma Treatments (Proteasome Inhibitors, Immunomodulatory Drugs (IMiDs).

Pharmacological treatment options for vertebral fractures include bisphosphonates, denosumab, teriparatide, and estrogen therapy. These medications have been shown to increase bone density and reduce the risk of subsequent fractures. In addition, bisphosphonates and denosumab have been shown to reduce the risk of vertebral fractures in patients with multiple myeloma.

Non-invasive treatments for vertebral fractures include bracing, physical therapy, and pain management. Bracing has been shown to improve pain and reduce the risk of further fractures in patients with acute vertebral fractures. Physical therapy can improve mobility and reduce pain in patients with chronic vertebral fractures. Pain management can be achieved through a variety of methods, including non-steroidal anti-inflammatory drugs (NSAIDs), opioids, and nerve blocks (1).

Interventional radiology plays a fundamental role in this pathology, especially in the treatment of pain associated with secondary vertebral fractures, mainly with the use of percutaneous kyphoplasty (KPT) and vertebroplasty (VPT) as indicated in 2017 CIRSE guidelines (1, 2).

Vertebral deformity and the development of adjacent level fractures at and above these osteoporotic fractures are significant long-term complications related to VPT and KPT (3, 4).

Although VPT allows a good reduction of pain both in the short and long term, normally does not allow an adequate restoration of the vertebral height and the kyphotic angle, often determining a vertebral deformity and alteration of the kyphotic angle which predisposes to adjacent vertebral fractures (5, 6).

Various Percutaneous Implant Techniques (PITs) were introduced in order to reduce the secondary loss of vertebral body height associated with PKP after balloon deﬂation and till cementation and to allow persistent restoration of vertebral height and restoration of a normal kyphotic angle (7).

Despite the literature supporting the efficacy of SpineJack [SJ] implant for treatment of vertebral post-traumatic compression fractures (8), no reports exist documenting its use in the treatment of compression fractures in multiple myeloma patients.

Our goal was to evaluate, for the first time, the feasibility and technical effectiveness of vertebroplasty with SJ implantation for the treatment of painful vertebral compression fracture, secondary to MM, to allow both an effective and prolonged pain reduction, restoration of vertebral height and ensure the biomechanical stability of the spine.

## Materials and methods

In this retrospective study, thirty- nine patients (19 women and 20 men; mean age, 64 with a range of 39–85 years) with MM who underwent VPT with SJ implantation between July 2017 and May 2022 were included (Table 1).

**Table 1:** Demographic characteristics of patients.

Patients’ characteristics	*n* (%)
Total Patients	39
Female	19 (48.7)
Male	20 (51.3)
Age (in years)	
Mean	64 ± 11.82
Range	39–85
Disease duration from initial diagnosis (in months)	
Mean	89.17 ± 19.11
Median	96.2
Range	3–204
Level of treated vertebrae	
T6-T11	23 (49.9)
T12-L2	11 (22.4)
L3-L5	15 (30.6)

A total of 49 vertebrae were treated with the implantation of 98 SJs.

The inclusion criteria were: back pain associated with the presence of a vertebral compressive fracture diagnosticated by a Computed Tomography (CT) scan or a Magnetic Resonance Imaging (MRI) scan.

The exclusion criteria were: extensive epidural and spinal canal infiltration (more than a third of the extension of the circumference of the epidural space), severe canal stenosis and moderate and severe neurologic deficits.

A systemic chemo-immunotherapy was previously performed to treat the neoplastic malignancy, and the vertebral augmentation was considered as a supportive therapy to reach a rapid relief of the pain.

All patients were previously treated with radiotherapy with persistent pain.

The pre-operative evaluation consisted of a combined oncological-radiological interventional clinical visit and the severity of pain was measured using the visual analog scale (VAS) and Functional Mobility Scale (FMS).

VAS score was evaluated at 1-week and 1- 3- 6-month follow-up.

FMS was recorded 1 month after the treatment to assess the effect of treatment on level of mobility and ability to walk. A 4-point FMS classification was used: 4, bedridden; 3, use of wheelchair; 2, limited painful ambulation; 1, normal ambulation.

### Technique

Vertebroplasty with SpineJack Implants was performed under dual CT (system: SOMATOM Sensation, Siemens, AG, Forchheim, Germany) and fluoroscopy guidance to monitor the correct visualization, advancement, and expansion of the implants, control potential posterior wall protrusion and monitor any leaks during cement injection.

All interventions were performed under conscious sedation with continuous intravenous infusion of fentanyl citrate (0.1 mg/2 ml diluted 1:10 with saline) and received local anesthesia with subcutaneous injection of lidocaine hydrochloride at 2% anesthesia.

Antibiotic prophylaxis (single dose of cefazolin, 2 g, intravenously) was performed in all patients.

Two 10-gauge bone trocars (Stryker) were inserted into the vertebral body via a bilateral transpedicular approach in the lumbar spine or via a costotransverse approach in the thoracic spine.

A blunt guide wire was placed bilaterally and through these a designed drill, mounted on a working cannula, was gently advanced manually and coaxially into the vertebral body, until the desired position of the implant to create the vertebral space for the implants.

After preparation of both sides, the two SpineJack® (Stryker Corp, Kalamazoo, MI) implants were inserted into the vertebral body and were gradually and simultaneously deployed until height restoration and kyphosis reduction were judged satisfactory.

After implant detachment Poly-Methyl-Methacrylate (PMMA) bone cement (SpinePlex® radiopaque bone cement - Stryker Corp, Kalamazoo, MI) was slowly injected, under real time fluoroscopy, through the same working cannula used to insert the implants into the vertebral body until satisfactory vertebral filling was obtained.

If leaks of cement appear, the procedure has been interrupted.

**Figure 1:**
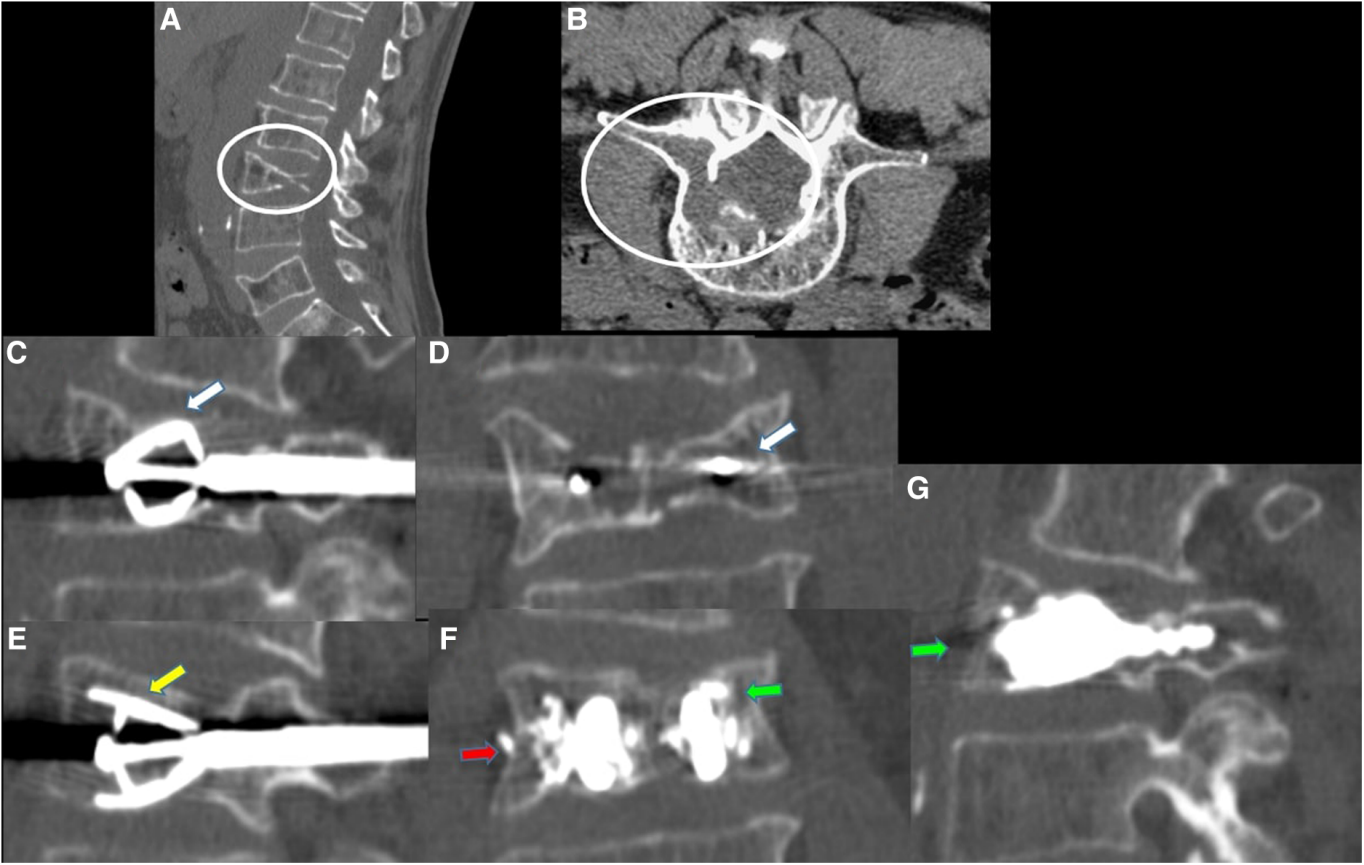
62-year-old male with a diagnosed MM with lumbar pain. A-B: Sagittal (**A**) and axial (**B**) CT scan showing L3 vertebra compression fracture, with significant reduction in the height of the posterior wall, with lytic lesion which also extends to the right pedicle [white circle in (**A,B**)]. (**C–E**): CT MPR (Multiplanar) in sagittal (**C–E**) and coronal (**D**) planes images; after preparation of both sides, two SpineJack® (white arrows), were inserted into the vertebral body and were gradually and simultaneously deployed [yellow arrow in (**E**)]. (**F–G)**: CT MPR in coronal (**F**) and sagittal (**G**)plane images; Post procedure control images showed a correct expansion of the vertebra with a homogeneous distribution of the vertebral cementum. Minimum right lateral cement leak was observed [red arrow in (**F**)].

**Figure 2:**
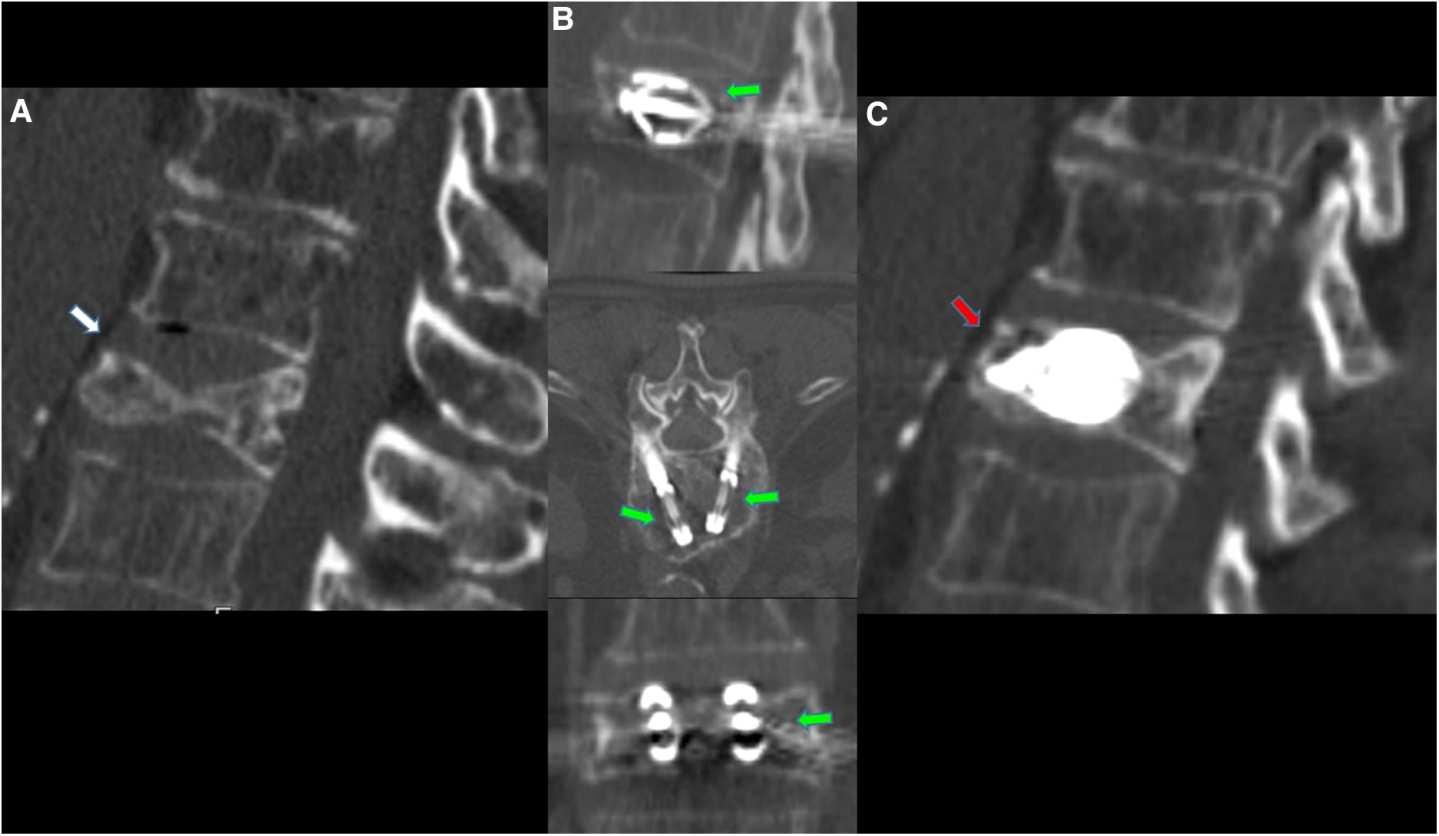
63-year-old male with a diagnosed MM with lumbar pain. (**A**) Sagittal CT scan in sagittal plane showing L2 vertebra compression fracture, with significant reduction in the height of the middle portion of vertebral body (white arrow). (**B**) CT MPR (Multiplanar) in sagittal, axial and coronal planes images; after preparation of both sides, two SpineJack® implants were inserted into the vertebral body and were gradually and simultaneously deployed (green arrows). (**C**) CT MPR in sagittal plane image; Post procedure control images showed a correct expansion of the vertebra with a homogeneous distribution of the vertebral cementum (red arrow). No cement leaks or complications.

**Figure 3:**
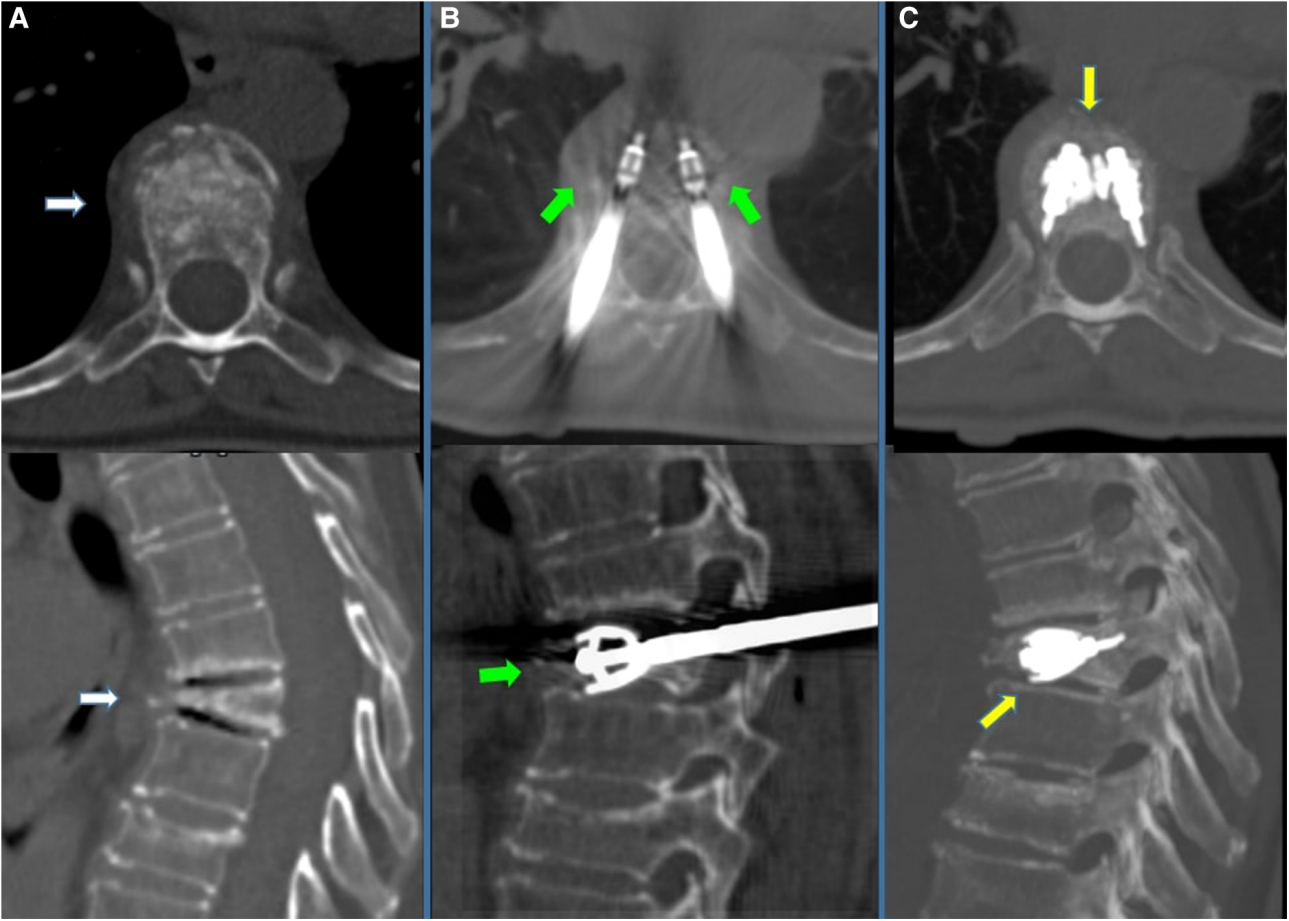
70-year-old female with a diagnosed MM with dorsal pain. (**A**) CT scan in axial and sagittal planes showing T6 vertebral compression fracture, with significant reduction in the height of the anterior wall (white arrows). (**B**) CT scan in axial and sagittal planes; after preparation of both sides, two SpineJack® implants were inserted into the vertebral body and were gradually and simultaneously deployed (green arrows). (**C**) CT scan using MIP (Maximum Intensity projection) reconstruction in axial and sagittal planes; Post-procedure control images showed a correct expansion of the vertebra with a homogeneous distribution of the vertebral cementum. No cement leaks or complications.

An immediate post-procedure no contrast-enhanced CT was performed to evaluate the results and any complications (Figures 1–3).

According to clinical follow-up, contrast-enhanced CT scans were acquired 6 months after the procedure.

### Statistical analysis

For the purposes of this study, continuous variables were shown as mean ± standard deviation (SD). Differences between the average VAS score at 1 week, 1, 3 and 6 months and FMS at 1 month after the procedure were evaluated by means of Student'st test or Fisher's exact test as appropriate. A *p* value less than 0.05 was taken as significant. Statistical analysis was performed using OpenStat software.

## Results

A total of 98 expandable Titanium SpineJack Implants were inserted into 49 vertebrae (49 bilateral procedures).

In 6 patients the Spinejack implants were implanted bilaterally in two vertebrae in the same operative session due to the presence of a double active fracture (Patients 2-4-7-20-21-34 in Table 2).

**Table 2:** Levels of the treated vertebrae, vertebral height variations,changes in VAS score and complications.

Patient	Vertebra	Anterior column height baseline	Anterior column height post	Middle column height baseline	Middle column height post	VAS baseline	VAS 1 week	VAS 1 month	VAS 3 months	VAS 6 months	Complications
1	L3	10 mm	13 mm	9 mm	13 mm	4	1	1	0	0	
2	D8	12 mm	15 mm	8 mm	12 mm	5	1	0	0	0	
	D9	12 mm	14 mm	10 mm	14 mm	5	1	0	0	0	
3	D8	13 mm	16 mm	21 mm	24 mm	5	1	1	1	0	
4	L2	13 mm	16 mm	13 mm	17 mm	5	3	0	0	0	
	L4	14 mm	14 mm	15 mm	17 mm	4	1	0	0	0	
5	D12	14 mm	17 mm	11 mm	16 mm	5	0	0	0	0	
6	L2	14 mm	14 mm	18 mm	22 mm	5	1	2	1	1	L3 vertebra fracture 7 days after L2 vertebra treatment
	L3	15 mm	16 mm	15 mm	19 mm	4	0	1	0	0	
7	D7	15 mm	17 mm	15 mm	20 mm	7	2	1	0	0	
	D9	15 mm	17 mm	15 mm	19 mm	6	1	2	1	1	
8	D12	16 mm	19 mm	12 mm	17 mm	7	3	0	0	0	
9	L4	16 mm	17 mm	12 mm	17 mm	5	0	0	0	0	
10	D6	17 mm	20 mm	13 mm	18 mm	5	2	1	0	0	
11	D12	18 mm	20 mm	14 mm	19 mm	7	3	2	0	0	
12	L2	18 mm	21 mm	17 mm	23 mm	6	2	2	1	1	
13	L4	18 mm	18 mm	14 mm	19 mm	6	3	0	0	0	
14	L4	19 mm	22 mm	14 mm	19 mm	6	0	0	0	0	Anterior venous cement leak
15	L2	21 mm	21 mm	18 mm	23 mm	6	1	0	0	0	
16	L3	21 mm	23 mm	16 mm	19 mm	6	1	1	1	0	
17	L1	21 mm	25 mm	18 mm	23 mm	6	1	0	1	0	
18	L3	23 mm	26 mm	16 mm	22 mm	5	1	0	0	0	
19	L1	23 mm	27 mm	18 mm	23 mm	4	0	0	2	1	L3 vertebra fracture 14 days after L1 vertebra treatment
	L3	23 mm	26 mm	22 mm	27 mm	4	1	0	0	0	
20	L3	23 mm	26 mm	20 mm	24 mm	6	4	3	1	2	L2 vertebra fracture 22 days after L3-D7 vertebrae treatment
	D7	24 mm	28 mm	18 mm	24 mm	5	4	1	1	1	
	L2	24 mm	26 mm	20 mm	24 mm	4	1	0	0	0	
21	D7	28 mm	30 mm	22 mm	26 mm	5	2	1	0	0	
	D9	10 mm	13 mm	9 mm	14 mm	5	1	0	2	1	D11 vertebra fracture 37 days after D7-D9vertebra treatment
	D11	6 mm	9 mm	8 mm	12 mm	7	1	1	0	0	
22	L4	12 mm	16 mm	10 mm	13 mm	6	2	0	0	0	
23	D10	13 mm	16 mm	21 mm	25 mm	6	1	0	0	0	
24	D10	14 mm	14 mm	15 mm	19 mm	4	1	0	0	0	
25	D7	13 mm	15 mm	13 mm	17 mm	7	2	2	1	1	Intradiscal cement leak
26	D9	14 mm	14 mm	11 mm	16 mm	6	0	1	0	0	
27	L1	14 mm	14 mm	18 mm	22 mm	4	0	0	0	0	
28	L5	15 mm	16 mm	15 mm	19 mm	6	0	1	0	0	
29	L3	15 mm	17 mm	15 mm	20 mm	6	2	1	0	0	Anterior venous cement leak
30	D10	15 mm	17 mm	15 mm	19 mm	6	2	0	1	0	
31	D12	16 mm	16 mm	12 mm	16 mm	8	3	3	1	0	
32	D10	16 mm	17 mm	12 mm	16 mm	5	1	0	0		
33	D11	17 mm	20 mm	13 mm	17 mm	5	2	1	0	0	Posterolateral leak
34	D9	18 mm	18 mm	17 mm	22 mm	6	2	1	0	0	
	L3	18 mm	20 mm	14 mm	19 mm	8	2	0	0	0	
35	L3	18 mm	18 mm	14 mm	18 mm	7	1	0	0	2	
36	D11	19 mm	22 mm	14 mm	18 mm	5	1	1	1	1	
37	D8	21 mm	21 mm	18 mm	22 mm	4	3	0	0	0	
38	D10	21 mm	23 mm	16 mm	19 mm	5	1	0	0	0	Intradiscal cement leak

The technical success rate was 100% (98/98) without major complications.

Minimal leakage of cement occurred in 5 procedures (10%), 2 anterior venous leakages, 1 posterolateral, and 2 intradiscal leakages, without clinical repercussions.

Four patients developed a secondary vertebral fracture in a caudal segment respectively 7,14,22 and 37 days after procedure.

The SJ procedure had a mean procedure duration of 23 ± 4 min.

Adjacent fractures were successfully treated with implantation of SpineJack implants (Patient 6,19,20 and 21 Table 2).

Vertebral height restoration was observed in 30 vertebrae (61%), with a mean anterior column height restoration of 2.0 mm (baseline 16.9 ± 4.3 mm vs. immediately postoperative 19.1 ± 3.6 mm *p* < 0.001); a mean middle column height restoration of 4.2 mm (baseline 14.9 ± 3.5 mm vs. immediately postoperative 19.1 ± 3.6 mm *p* < 0.001).

All patients were discharged 6–8 h after treatment in stable and uncomplicated conditions.

No patients were lost to follow-up at 6 months.

Mean VAS score of pain evaluation on the day before treatment was 5.4 ± 1.0 (range 4–8).

**Figure 4:**
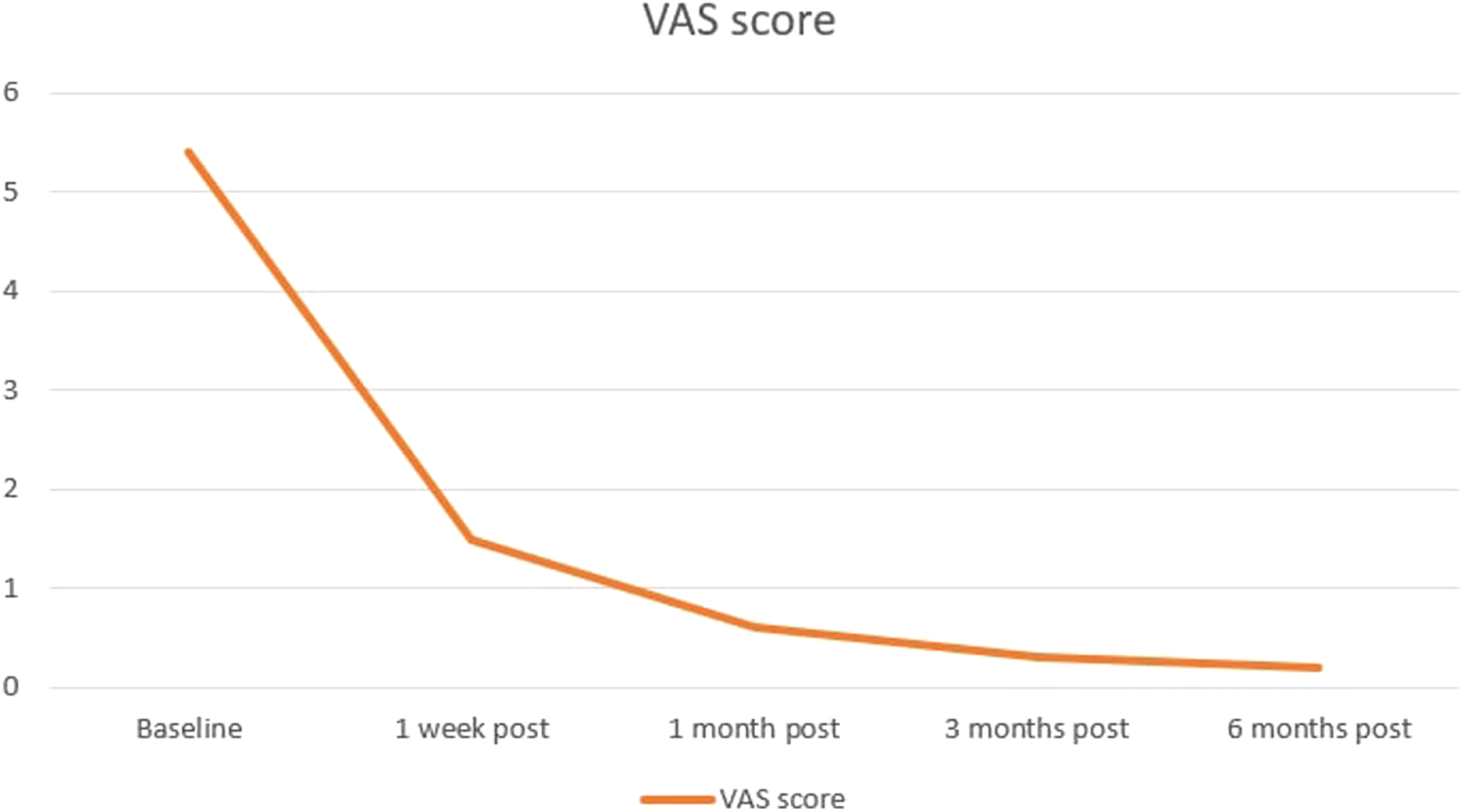
Median VAS score follow-up evaluated before and prospectively after 1 week, and 1, 3, and 6 months from the treatment. Orange line indicates patients’ changes in pain.

One week after treatment the median VAS score of pain was 1.5 ± 1.2 (range, 0–6) with a mean reduction of 72.2% (5.4 ± 1.0 vs. 1.5 ± 1.2; *p* < 0.000; Figure 4).

One month after treatment the median VAS score of pain was 0.6 ± 0.8 (range, 0–3) with a mean reduction of 88.89% (5.4 ± 1.0 vs. 0.6 ± 0.8; *p* < 0.000; Figure 4) compared with baseline evaluation.

At the 3-month evaluation, the median VAS score for pain was 0.3 ± 0.5 (range 0–2) with a mean reduction of 94.44% (5.4 ± 1.0 vs. 0.6 ± 0.8; *p* < 0.000;Figure 4) compared with baseline evaluation.

At the 6-month evaluation, the median VAS score for pain was 0.2 ± 0.5 (range 0–2) with a mean reduction of 96.3% (5.4 ± 1.0 vs. 0.2 ± 0.5; *p* < 0.000;Figure 4) compared with baseline evaluation.

The levels of the treated vertebrae, vertebral height variations, VAS scores and complications are described in Table 2.

Mean FMS on the day before treatment was 2.3 ± 0.5. (range 2–4).

One month after treatment the median FMS of disability was 1.2 ± 0.4 (range, 1–3) with a mean reduction of −47.8% (2.3 ± 0.5 vs 1.2 ± 0.4; *p* < 0.000;) compared with baseline evaluation.

During follow-up, no infectious complications were observed.

Contrast-enhanced CT or MRI scans performed 6 months after the procedure showed no local recurrence, implant displacement or new fractures in the treated site.

## Discussion

Our study demonstrates that SpineJack implant followed by VPT is a feasible and safe procedure that allows to reduce back pain due to vertebral involvement in MM and at the same time allows to re-establish vertebral height, allowing persistent spinal stability.

MM in 90% of cases presents bone involvement and frequently affects the vertebral column and causes vertebral fractures and collapses, acute pain and possible associated secondary neurological deficit (9).

Although it is a highly radiosensitive tumor and radiotherapy often allows a significant reduction of pain, it does not allow the restoration of the vertebral height and the correct kyphotic angle, and the consequent deformity of the spine can increase mortality and morbidity associated with this disease (10).

Stereotactic radiotherapy (SR) is also associated with up to 18% of adjacent post-treatment vertebral fractures (11).

Kado et al. showed that in older women with vertebral fractures, hyperkyphosis predicts increased risk of death, independent of underlying spinal osteoporosis and the extent and severity of vertebral fractures (12).

As evidenced on human specimen models, endplate depression after associated with an osteoporotic vertebral fracture, impairs the ability of the disc to distribute load evenly to the adjacent segments.

Load concentration on the anterior portion of the adjacent vertebrae may contribute to increased subsequent fracture risk after an osteoporotic vertebral fracture which may be associated with increased morbidity and mortality in these patients (13).

This secondary alteration of the biomechanics of the spine can be reduced by a realignment of the vertebral endplates, restoring normal disk mechanics and load sharing, through KPT or PITs (14).

Since 1996 percutaneous VPT (15) and since 2002 KPT (16) have been used in the treatment of vertebral fractures associated with MM with good results in terms of pain reduction and with greater efficacy of KPT in the prevention of early adjacent fractures.

In a recent systematic review and meta-analysis, which retrospectively evaluated about 2 million patients, it was confirmed that the execution of VPT and KPT reduced by at least 22% the 10-year mortality after compression fracture.

KPT provided mortality benefits over VPT, with reported hazard ratios of 0.77 (95% CI: 0.77, 0.78; *p* < .001) and 0.87 (95% CI: 0.87, 0.88; *p* < .001), respectively (17).

Consensus statement from the International Myeloma Working Group (IMWG) concluded that “Cement augmentation is a very effective way of stabilizing the anterior and middle spinal columns without the need for metalwork fixation” (18).

In 2017 CIRSE guidelines, VPT, KPT and percutaneous implant techniques (PIT) are indicated in the treatment of painful vertebrae with extensive osteolysis due to malignant infiltration by multiple myeloma, lymphoma and metastasis (2).

Despite the good short-term efficacy, various studies have highlighted two main problems in the follow-up of KPT: loss of the initial vertebral height correction with subsequent angulation and an increased incidence of fractures, mostly at the superior adjacent vertebra (19–21).

For this reason it appears necessary to evaluate the use of vertebral implants as a therapeutic option since they allow an effective and persistent reduction of the deformity.

SpineJack (Stryker, Kalamazoo, Michigan) is an expandable titanium intravertebral implant that restores vertebral height and maintains a correct kyphotic angle of the spine resulting in a more balanced distribution of axial load on fractured vertebrae, a restoration of intervertebral disc function, both in the pathological segment and throughout the spine (22).

Its use has been mainly studied in the treatment of acute vertebral compression fractures with good results in terms of pain reduction and preservation of spine stability in short and long-term follow-up (23–27).

Comparative studies between SJ and KPT have shown that the use of SJ is associated with less spinal deformity; the incidence of adjacent fractures consequently is significantly lower when using the SJ (3% to 5%) than 15%–20% with KPT (28, 29).

While SpineJack has been shown to be effective in the treatment of vertebral compression fractures, there are potential complications associated with the procedure.

One potential complication is cement leakage, which occurs when the bone cement used to stabilize the fracture leaks into surrounding tissues.

However, studies have shown that the incidence of cement leakage with SpineJack is relatively low compared to other vertebral augmentation procedures (23–27).

Another potential complication of SpineJack is vertebral refracture, which can occur when the treated vertebrae are subjected to additional stress and fracture again. The risk of refracture can be reduced with appropriate patient selection criteria and post-procedure rehabilitation.

In addition, there is a risk of infection with any invasive procedure, including SpineJack.

Other potential complications associated with SpineJack include bleeding, nerve injury, and device failure. However, these complications are rare and can usually be managed through appropriate monitoring and intervention (23–29).

Only one study has been published that evaluated the use of the SJ in the treatment of vertebral pathological compression fractures secondary to bone metastases, with good results in terms of pain relief and reduction of complications (30).

To date, this is the first study analyzing the efficacy of SJ in the treatment of vertebral fractures secondary to MM.

Our preliminary experience coincides with the study by Cornelis et al. (30) highlighting a good reduction in pain associated up to 6 months with an excellent recovery of physical activity.

Furthermore, vertebral height was significantly increased both anteriorly and centrally a homogeneous cement distribution with a small and not clinically significant percentage of cement leaks.

We believe that the advantage of using the combined CT-Fluoroscopy technique is especially evident in patients in whom the spine has significant pre-existing deformity and the CT guide has allowed us easier access to the vertebra and greater precision in positioning the implants.

The fluoroscopic guidance allowed us a real-time synchronous opening of the implants, with excellent control of the restoration of the vertebral height, the preservation of the posterior wall and finally the correct distribution of the cement (Supplementary Video S1).

An important factor was the use of mild sedation which allowed a rapid postoperative recovery of the patient, control of vital functions and a consequent rapid discharge, significantly reducing hospitalization times.

We also believe that this innovative methodology could represent a further therapeutic option to be taken into consideration in some cases, selected in a multidisciplinary context, even before radiotherapy, in order to prevent post-irradiation fractures.

Our study has limitations, being a single-center, retrospective study based on a small cohort of patients and short follow-up.

Prospective comparative studies with long follow-up between the use of KPT and SJ in patients with MM are also needed to highlight the effective superiority of SJ in terms of reduction of complications secondary to spine instability.

Nevertheless, our preliminary study shows that this technique is feasible and safe also in the treatment of vertebral compression fractures secondary to MM with a good reduction of pain, restoration of mobility and increase of spinal stability.

## Conclusions

This preliminary study highlights that the use of bilateral expandable titanium SpineJack implants, followed by vertebroplasty, is a safe and effective procedure for the treatment of vertebral fracture from pathological compression secondary to MM, allowing an adequate restoration of the vertebral height and a correct distribution of the craniocaudal load forces on the vertebral column.

We observed a rapid and persistent improvement from the pain, resulting in a rapid improvement in the patient's mobility and ambulation.

## Data Availability

The raw data supporting the conclusions of this article will be made available by the authors, without undue reservation.

## Supplementary material

SUPPLEMENTARY VIDEO S1

70-year-old female with a diagnosed MM with dorsal pain.Fluoroscopic stages of insertion, opening and cementation of jack plug implants.
